# Identifying predictive hematological biomarkers for radiation exposure by machine learning in mouse models

**DOI:** 10.1038/s43856-026-01690-8

**Published:** 2026-06-19

**Authors:** Hang Chang, Yiyan Yao, Jared DeChant, Lieselotte Obst-Huebl, Kenneth H. Wan, Soo Park, William Fisher, Kei Nakamura, Susan E. Celniker, Antoine M. Snijders, Jian-Hua Mao, Jamie L. Inman

**Affiliations:** 1 Biological Systems and Engineering Division, Lawrence Berkeley National Laboratory, 1 Cyclotron Rd, Berkeley, CA USA; 2 Berkeley Biomedical Data Science Center, Lawrence Berkeley National Laboratory, Berkeley, CA USA; 3 Accelerator Technology and Applied Physics Division, Lawrence Berkeley National Laboratory, 1 Cyclotron Rd, Berkeley, CA USA; 4 Present Address: Biosciences & Biotechnology Division, Lawrence Livermore National Laboratory, Livermore, CA USA

**Keywords:** Diagnostic markers, Predictive markers

## Abstract

**Background:**

Population-scale radiation exposure assessment during radiological emergencies is hindered by the slow and costly nature of current methods, creating a need for rapid, affordable screening tools. Radiation biodosimetry using peripheral blood counts is a promising approach, but estimating low-dose exposures and exposure at extended time points remains challenging, especially when accounting for inter-individual differences in radiation sensitivity.

**Methods:**

We analyze complete blood count (CBC) profiles from a retrospective cohort of 1151 male and female BALB/cJ and C57BL/6 J mice exposed to total-body X-ray radiation at doses ranging from 0.05 to 4 Gy. CBCs are collected 1 to 150 days post exposure. We develop a predictive model of radiation exposure using a sparse representation learning strategy to identify the most informative CBC parameters. Model performance is evaluated through exhaustive cross-validation and validated in a double-blind prospective cohort of 431 animals. To evaluate robustness in a genetically diverse population, we further test the model on CBC data from a Collaborative Cross (CC) cohort of 1720 animals representing 35 CC strains, 24 h and 28 days after sham or 1 Gy total-body X-ray exposure.

**Results:**

Exhaustive cross-validation shows good performance of the Sparse CBC model, with AUC, accuracy and sensitivity exceeding 80%. Similar performance is observed in the prospective cohort. In the CC cohort, performance is modest. Importantly, model performance varies across CC strains, suggesting that host genetic background significantly influences predictive accuracy.

**Conclusions:**

Our findings demonstrate that the Sparse CBC model effectively leverages CBC data to estimate radiation exposure across multiple mouse cohorts, including genetically diverse CC populations. While CBC-based predictions provide a complementary tool for exposure assessment, model performance varies with genetic backgrounds.

## Plain language summary

During radioactive exposure emergencies, rapidly identifying who has been exposed to radiation is essential for providing timely medical care. Current methods are often expensive, and unscalable. Routine complete blood count (CBC) tests offer a potential alternative because radiation alters blood cell numbers and composition. In this study, we analyzed CBC data from more than 1100 mice over periods up to 150 days after radiation exposure. Using these data, we developed a computer program that identifies the most informative blood cell measurements for detecting radiation exposure. The program performed well across multiple, but performance varied across genetically diverse mouse strains, highlighting the influence of genetic background.

## Abstract (web summary)

Chang et al. describe a machine-learning model using routine complete blood count tests to detect radiation exposure across doses and time points, enabling rapid identification of radiation exposure is essential during nuclear emergencies. The model performs well but varies across genetic backgrounds, highlighting the role of genetics in radiation response.

## Introduction

Development of efficient radiation biodosimetry tools is crucial for public health for several reasons, including timely and accurate emergency response, public health monitoring, improved medical triage, risk assessment and personalized medicine. The current gold standard in radiation biodosimetry uses cytogenetic measurements of chromosomal abnormalities in metaphases of cultured lymphocytes^1,2^. This method has played an important role in estimating radiation dose in atomic-bomb survivors in Hiroshima and Nagasaki, Japan^3^, individuals exposed after the Chornobyl nuclear^4–6^, and in the Fukushima Daiichi accident^2,7,8^. Biochemical biomarkers of radiation exposure have been developed as an alternative or supplementary method for detecting radiation exposure^9^. They include transcriptomic and proteomic biomarkers that are activated as part of the damage response pathways after radiation exposure, including DNA repair, cell cycle functions and apoptosis^10^.

Despite these advances, current radiation biodosimetry methods have several drawbacks, including complexity, cost, long turnaround times and limited sensitivity and specificity. Many biodosimetry techniques require specialized equipment and trained personnel, making them expensive and logistically challenging to implement in emergencies. Some methods, such as cytogenetic assays, require several days to yield results, delaying time-sensitive clinical decision-making. Existing tools also do not always reliably detect low-dose radiation (<1 Gy) exposure or may produce false positives/negatives, which complicates exposure assessment. In addition, individual responses to radiation can vary significantly based on genetic factors, health status, and age, complicating the interpretation of biodosimetry results. Together, these limitations highlight the need for rapid, accessible, and robust biodosimetry tools that perform well across diverse populations.

The CBC, a widely available clinical test, is a cornerstone in evaluating and managing radiation exposure. CBC parameters are acutely affected by radiation exposure, as radiation rapidly divides cells of the hematopoietic compartment, which are particularly sensitive to radiation. As such, clinicians incorporate CBC values in the assessment of acute radiation syndrome severity, progression, recovery and risk of complications^11^. CBC-based approaches also support monitoring of radiation workers,^12^;however, the CBC alone is not generally considered a valid stand-alone biodosimetry test for most occupational exposures. Broader use of the CBC in this context remains limited by the relatively small number of dedicated studies and by confounding factors, including population-level genetic diversity in CBC parameters^13,14^.

Mouse models provide important advantages for investigating the usefulness of CBC tests in predicting radiation exposure because they allow controlled testing across defined exposure conditions and biological backgrounds. At the same time, advances in artificial intelligence (AI) and machine learning (ML) methods offer an avenue to explore the use of the CBC in biodosimetry workflows and present opportunities to apply cross-species analytical approaches^15,16^. ML models on CBC parameters have been used for many different disease states. For example, investigators apply ML for differentiation among leukemias,^17^ to support early prediction of bacteremia^18^, to aid COVID-19 detection,^19^ to inform diagnostic test decision making^20^ and to improve clinical risk evaluation^13^. These developments support the rationale for investigating whether CBC-based AI/ML approaches can improve radiation biodosimetry.

In this study, we develop and evaluate an AI/ML model to predict sublethal and low-dose radiation exposure using CBC parameters. We evaluate the model across multiple murine cohorts and demonstrate that CBCs provide predictive value at doses as low as 0.05 Gy and at time points up to 150 days post-exposure. These findings establish CBC-based AI models as a complementary, rapid, and scalable tool for radiation biodosimetry.

## Materials and methods

### Animal cohorts

#### C57BL6/J and BALB/cJ cohorts

Mice were obtained from Jackson Laboratories and acclimated at Lawrence Berkeley National Laboratory (LBNL), maintained under standard pathogen-free conditions. All blood collections were performed between 1 and 5 p.m. All procedures were approved by the Institutional Animal Care and Use Committee of LBNL and the Animal Welfare and Research Committee, under approved protocol number 270032, and complied with NIH guidelines and ARRIVE 2.0.

#### Population-based CC cohort

is described in He et al.^14^. In brief, mice were obtained from the Systems Genetics Core Facility at The University of North Carolina at Chapel Hill^21^. Mice were acclimated at LBNL for 8 weeks prior to the initiation of breeding as previously described^22^. For each strain, 12-week-old mice were exposed to 1 Gy or sham irradiation, and blood was collected at 24 h or 28 days post-irradiation.

### Radiation exposure

Whole-body X-ray irradiation or sham treatment was delivered using a Precision X-ray Inc XRAD320 320 kVp X-ray machine, operated at 300 kVp, 2–10 mA. Animal cages were randomly assigned to irradiation dose groups. The dose rate in the retrospective cohort was 0.18 Gy/min for 0.05, 0.1, 0.5 and 1 Gy treatments and 1.3 Gy/min for 2 and 4 Gy treatments. The dose rate in the prospective validation cohort was 1.3 Gy/min, and the dose rate in the population-based CC cohort was 0.196 Gy/min. A hardening filter consisting of 0.5 mm Cu was used, which provides ~3 mm HVL Cu. Mice were not anesthetized and were placed in a plastic holder and irradiated on a rotating stage. Two animals in individual holders were placed side by side in the center of the field. Dosimetry was performed using a RadCal ion chamber and radiochromic film demonstrating uniformity within 4% across the exposure field^23^.

### CBC

For all cohorts, whole blood was collected into EDTA-coated tubes (Fisher Scientific). A CBC was acquired within 30 minutes of collection using a HemaVet950FS. All cohorts were analyzed on the same machine.

### Model construction

#### Random forest

Random forest machine learning model was deployed with default parameter settings as (1) the baseline model using original CBC features; and (ii) the classifier based on Sparse CBC features to demonstrate the effectiveness of Sparse CBC over raw CBC. Model discrimination was summarized by AUC. For calculation of accuracy, sensitivity and specificity, predicted probabilities were converted to binary class labels (irradiation: Yes/No) using a prespecified threshold of 0.5, selected a priori and not optimized post hoc.

#### Sparse CBC

We extended the unsupervised feature learning pipeline (SPSD) (16,17), to realize efficient and effective mining of CBC signatures. Given $X=[{x}_{1},\ldots ,{x}_{N}]\in {R}^{m\times N}$ as a set of mice (N) with CBC phenotypes, the formulation of the Sparse CBC model is defined as follows.

$$\it {{{\min }}}_{{{\rm{ B}}},{{\rm{ Z}}},{{\rm{ W}}},{{\mathrm{G}}}}{{||}{{\mathrm{X}}}-{{\rm{BZ}}}{||}}_{{{ F}}}^{{{2}}}+{{||}{{\mathrm{Z}}}-{{\mathrm{G}}}{{\sigma }}({{\rm{WX}}}){||}}_{{{ F}}}^{{{2}}}+{{{\lambda }}}_{{{1}}}{{||}{{\mathrm{Z}}}{||}}_{{{1}}}$$

$${{\boldsymbol{s}}}.{{\boldsymbol{t}}}.{{||}{{{\boldsymbol{b}}}}_{{{\boldsymbol{i}}}}{||}}_{{{\bf{2}}}}^{{{\bf{2}}}}={{\bf{1}}},\forall {{\boldsymbol{i}}}={{\bf{1}}},\ldots ,{{\boldsymbol{h}}}$$

where $B=[{b}_{1},\ldots ,{b}_{h}]\in {R}^{m\times h}$ is a set of CBC signature to be mined. Each signature (b) is composed of *m* individual phenotypes (e.g., *m* = 20 in our study); $Z=[{z}_{1},\ldots ,{z}_{N}]\in {R}^{h\times N}$ is the sparse signature expression matrix, where ${z}_{i}$ is the sparse signature expression profile of the original mice CBC phenotypes (${x}_{i}$), consisting of relative abundances of all (h) signatures that contribute to the reconstruction of ${x}_{i}$; $W{\in R}^{h\times m}$ is the auto-encoder for efficient and effective extraction of sparse signature expression matrix (Z) from original mice CBC phenotype data (X); $G={diag}({g}_{1},..,{g}_{h})\in {R}^{h\times h}$ is a scaling matrix with diag being an operator aligning vector, $[{g}_{1},..,{g}_{h}]$, along the diagonal; $\sigma \left(\cdot \right)$ is an element-wise sigmoid function; ${\lambda }_{1}$ is the regularization constant to ensure the sparsity of Z, such that only a subset of signatures will be utilized during the reconstruction of original mice CBC phenotype data.

The first constraint: ${{||X}-{BZ||}}_{F}^{2}$, penalizes the reconstruction error of original mice CBC phenotype data (X) with CBC signature (B) and the corresponding sparse signature expression matrix (Z), which helps minimize the loss of individual signature information; the second constraint: ${{||Z}-G\sigma ({WX}){||}}_{F}^{2}$, penalizes the approximation error of sparse signature expression matrix (Z) with the auto-encoder, which helps improve the accuracy of signature extraction for new mice; the third constraint: ${{||Z||}}_{1}$, penalizes the sparsity of the signature expression matrix, which helps ensure the utilization/activation of dominant signatures during the learning process.

### Radiation exposure score

The Radiation Exposure Score is the predicted probability output of the trained Random Forest classification model for a given dose and time point. This score is not derived from a closed-form equation; rather, it reflects the model-inferred likelihood of radiation exposure based on learned relationships within the CBC feature space. Reproduction of the Radiation Exposure Score for new datasets is enabled through the publicly available analysis code and trained model objects described in the Data Availability section.

### Statistical analysis

All statistical analyses were performed in R (version 4.0.2, https://www.r-project.org/) Graphic visualizations were generated using R (ggplot2 package, Version 3.3.3; ggpubr package, Version 0.4.0). Box and whisker plots indicate median, 25 and 75th percentiles, and individual samples beyond these limits. Outliers are cases with values beyond 1.5 times the interquartile range and are shown for visualization purposes only; no samples were excluded as outliers from any analyses. *P*-values were obtained using a two-sided Mann–Whitney test for comparison of continuous variables between groups: ns (not significant), *(*p* < 0.05), **(*p* < 0.01), ***(*P* < 0.001), ****(*P* < 0.0001). Generalized Linear Models were used to assess differences in radiation exposure scores across doses and time points post-exposure.

## Results

### Study design and animal cohorts

**Fig. 1:**
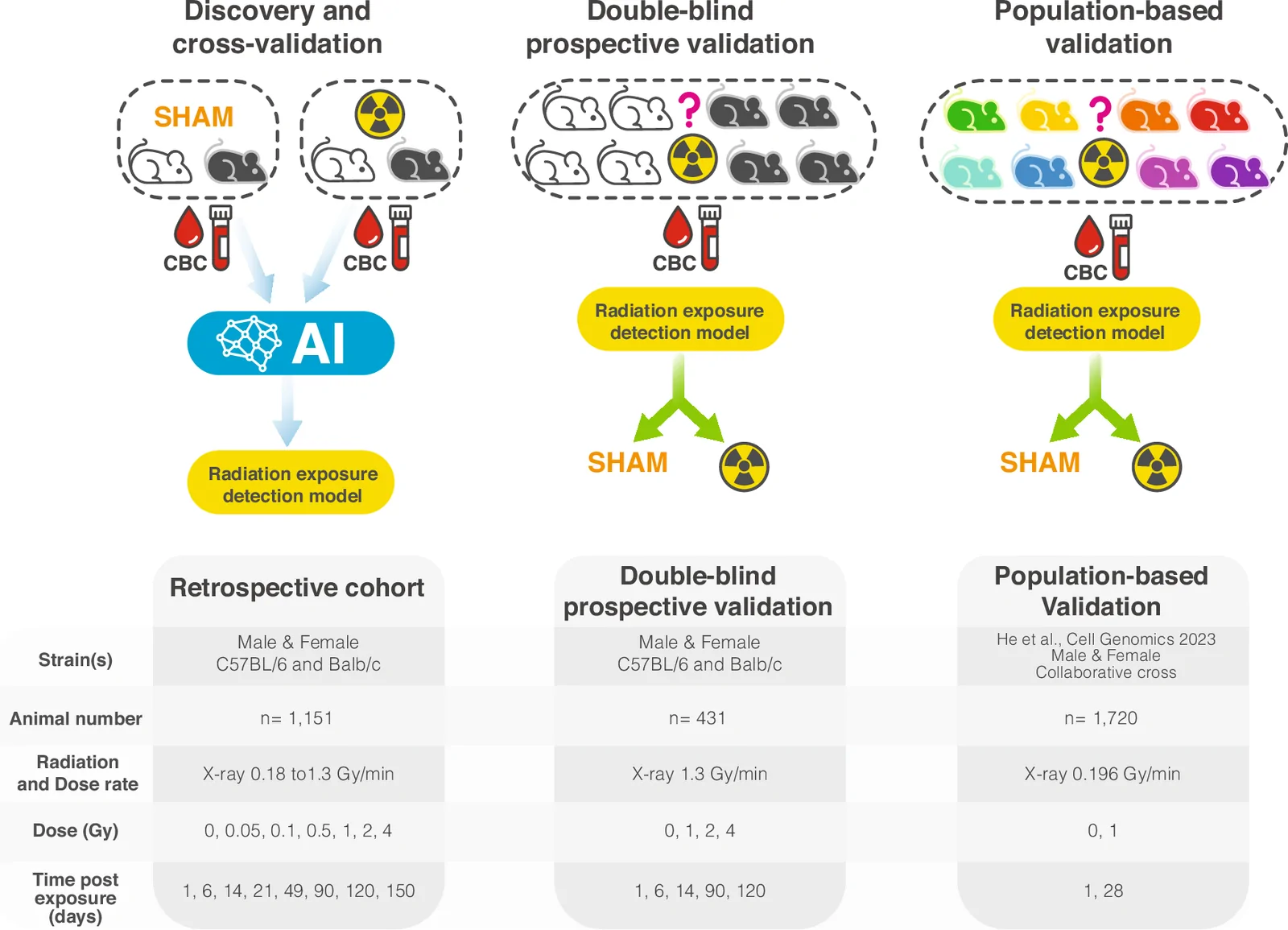
Overview of experimental design. Cohort characteristics of the three cohorts studied are depicted: a discovery and cross-validation cohort of 1151 mice, a double-blind prospective validation cohort of 431 mice, and a population-based validation cohort of 1720 mice.

Our study analyzed three distinct mouse cohorts to develop and validate our predictive model. The retrospective cohort used for model training included 1151 mice that received a single total-body irradiation (TBI) dose ranging from 0.05 Gy to 4 Gy or sham treatment. Blood was collected at various time points from 1 to 150 days post-irradiation, and CBC data were compiled from multiple previous studies (Fig. 1; Table S1). CBC data from the retrospective cohort (1151 mice) were used to train the model and assess its accuracy and generalizability via cross-validation. The dose rate in the retrospective cohort was 0.18 Gy/min for animals treated with 0.05, 0.1, 0.5 and 1 Gy. The dose rate was 1.3 Gy/min for animals treated at 2 and 4 Gy. The prospective validation cohort, used to evaluate model performance under double-blinded conditions, consisted of 431 mice that received a single TBI dose ranging from 1 Gy to 4 Gy at 1.3 Gy/min or sham treatment, with blood collected at similar time points (Fig. 1; Table S2). The CC validation cohort provided an additional layer of validation using genetically diverse mice. This cohort included 1720 CC mice from 35 strains^14^, exposed at 12 weeks of age to 1 Gy whole-body X-ray radiation at 0.196 Gy/min or sham treatment. Blood was collected either 24 h (444 sham, 355 exposed) or 4 weeks (453 sham, 468 exposed) after exposure (Fig. 1; Table S3). To minimize confounding factors, all irradiations were performed between 1:00 and 2:00 p.m., and all blood collections occurred between 1:00 and 5:00 p.m.

### Hematologic responses to radiation exposure depend on doses and time post exposure

**Fig. 2:**
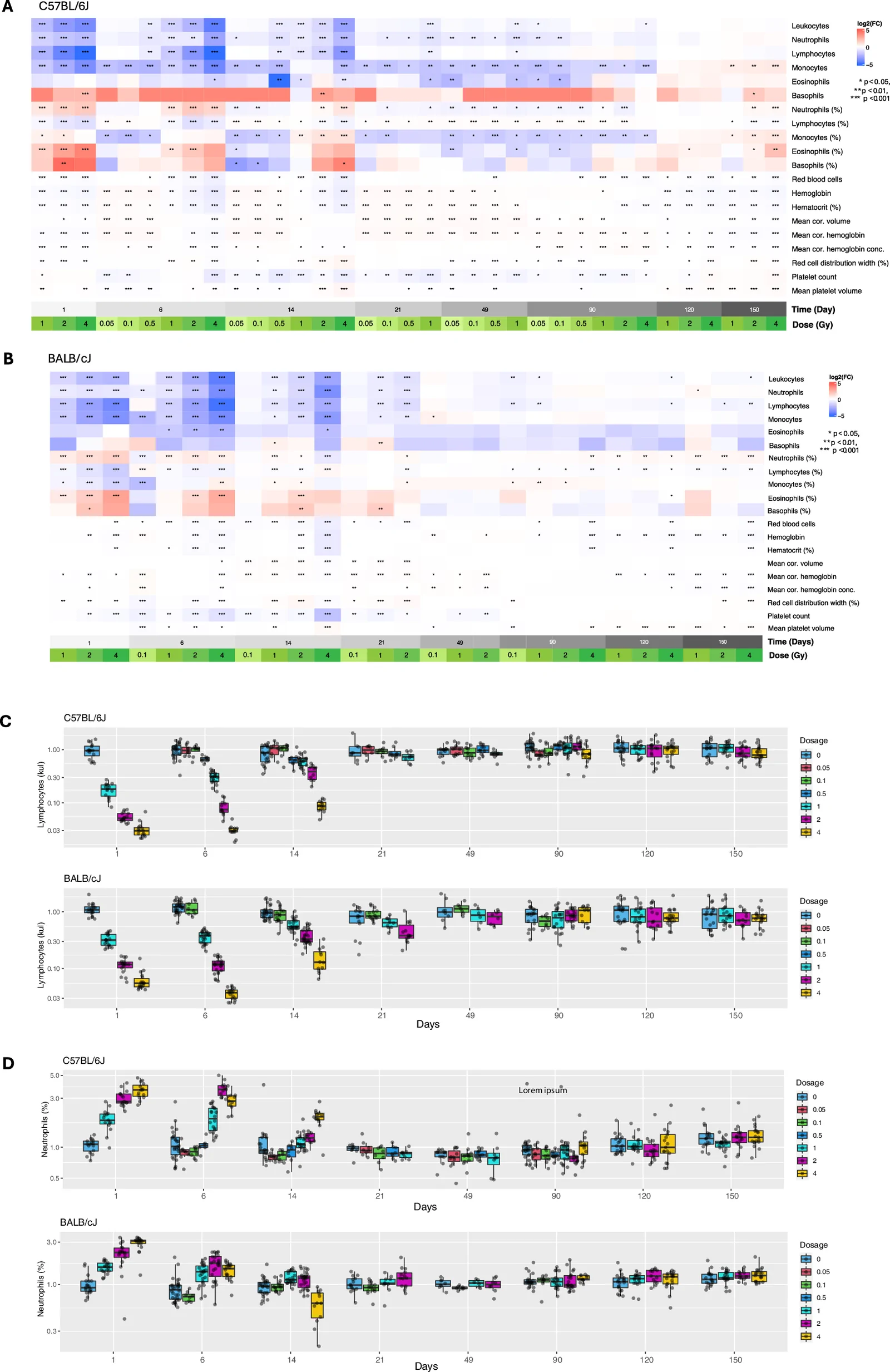
Heatmaps and relative lymphocyte counts and neutrophil percents. **A** Heatmap of changes in CBC parameters in C57BL/6 J CBC counts post-exposure in the Retrospective Cohort. Dose is indicated on the bottom in green (0.05-4 Gy) and time post exposure in gray (1–150 days). The p values were obtained using two-sided Mann-Whitney U tests. No adjustment was applied for multiple corrections. Significance for each metric is indicated in the heat graph with *p < 0.05, **p < 0.01, ***p < 0.001. **B** Heat map of changes in CBC parameters in Balb/cJ CBC counts post-exposure in the Retrospective Cohort. Dose is indicated on the bottom in green (0.1-4 Gy) and time post exposure in gray (1–150 days). Significance for each metric is indicated in the heat graph with *p < 0.05, **p < 0.01, ***p < 0.001. **C** Relative lymphocyte counts (kul) in C57BL/6 J (top; *n* = 573) and Balb/cJ (bottom; *n* = 578) over time post exposure. **D** Relative neutrophil % in C57BL/6 J (top; *n* = 573) and Balb/cJ (bottom; *n* = 578) over time post exposure. Box and whisker plots indicate median, 25 and 75th percentiles, and individual samples beyond these limits.

We investigated how radiation dose and time post-exposure influence hematologic responses by analyzing a comprehensive 20-parameter CBC profile using an automated hematology system. A cohort of 1,151 mice, aged 9-12 weeks, was exposed to whole-body X-ray radiation at doses of 0 (sham), 0.05, 0.1, 0.5, 1, 2, or 4 Gy. Blood samples were collected at multiple time points following exposure - 1, 6, 14, 21, 49, 90, 120, and 150 days - for CBC analysis (Fig. 1). We observed that dose-response patterns varied across different blood cell types and were influenced by both time post-exposure and genetic background (C57BL/6 J vs. BALB/cJ) (Fig. 2; Fig. S1). For instance, lymphocyte counts showed a significant dose-dependent decrease at early time points (≤21 days) in both strains. However, while lymphocyte levels recovered over time in C57BL/6 J mice (Fig. 2C; Fig. S1), they remained suppressed in BALB/cJ mice, particularly at higher radiation doses. Additionally, percent neutrophils increased significantly with dose in BALB/cJ mice and remained elevated for a longer duration compared to C57BL/6 J mice (Fig. 2D; Fig. S1). We conclude that genetic backgrounds and time post-exposure significantly influence the dose-response of blood cells to whole-body X-ray exposure.

### Radiation-exposure predictive model construction and cross-validation on retrospective cohort

**Fig. 3:**
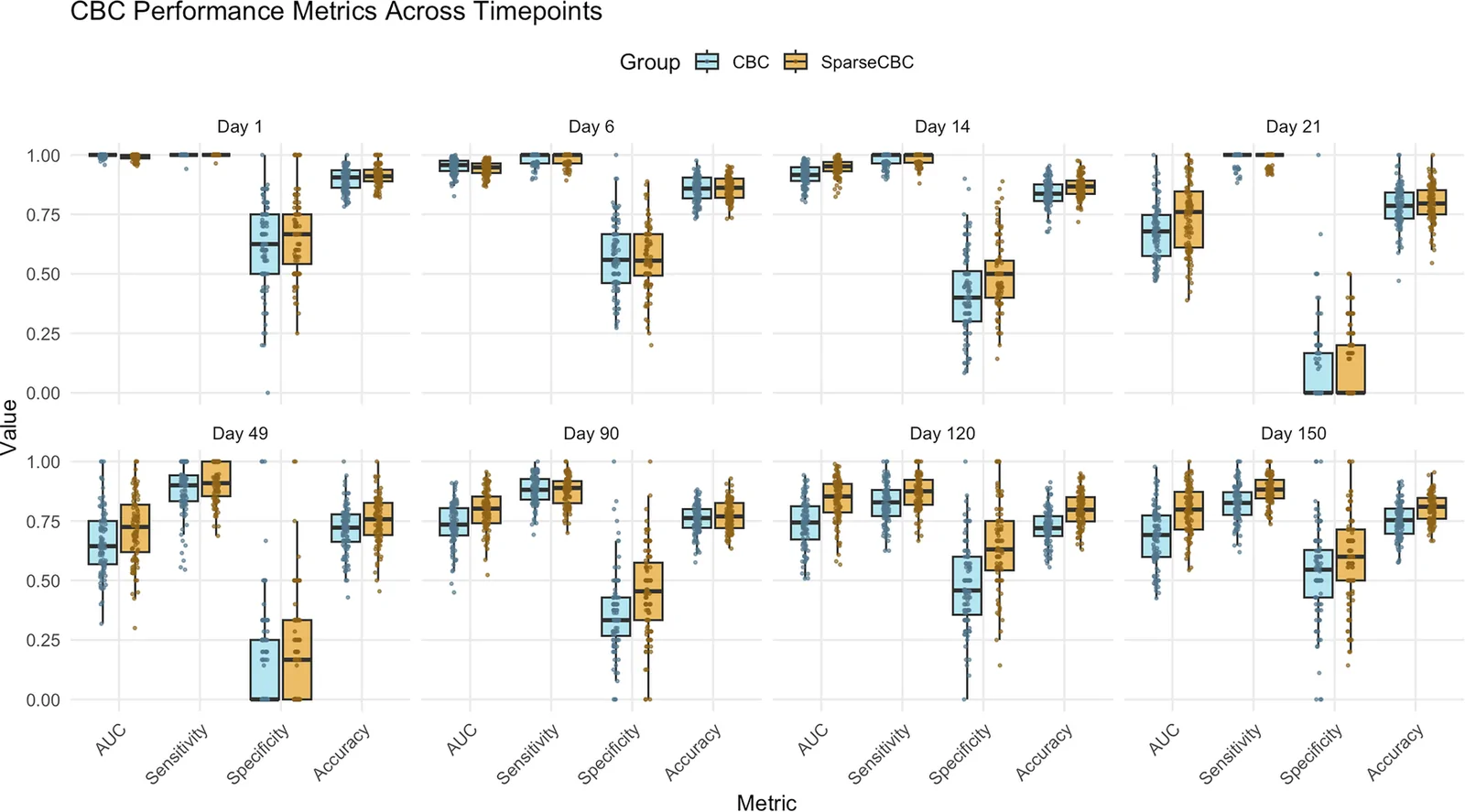
Model Construction Retrospective Cohort. Boxplots show AUC, sensitivity, specificity and accuracy for the full CBC and Sparse CBC models at days 1, 6, 14, 21, 49, 90, 120 and 150 post exposure. All individual data points are overlaid on the boxplots *n* = 1151. The p values obtained using two-sided Mann-Whitney U tests are provided in Table S4. Box and whisker plots indicate median, 25th and 75th percentiles, and individual samples beyond these limits.

To develop a predictive model for detecting radiation exposure, we applied the Sparse CBC technique to the retrospective cohort, transforming 1151 CBC profiles into a sparse feature space that captures essential immune signatures. We then trained a random forest classification model on both the original CBC features and the Sparse CBC features using a bootstrapping strategy with 100 iterations and a 60% sampling rate. The Sparse CBC model significantly outperformed the model based on original CBC features in predicting irradiation status (*p* < 0.05), with improved performance metrics: AUC = 0.870 [95% CI: 0.864, 0.875], Accuracy = 0.829 [95% CI: 0.827, 0.831], Sensitivity = 0.942 [95% CI: 0.934, 0.945], and Specificity = 0.509 [95% CI: 0.491, 0.526], compared to AUC = 0.826 [95% CI: 0.821, 0.833], Accuracy = 0.797 [95% CI: 0.792, 0.801], Sensitivity = 0.924 [95% CI: 0.916, 0.932], and Specificity = 0.448 [95% CI: 0.424, 0.469] for the original CBC features (Fig. S2). When performance was assessed across individual time points, the Sparse CBC model consistently outperformed the original CBC model starting from day 14 through day 150 post-exposure (Fig. 3, Table S4), demonstrating enhanced ability to detect radiation exposure at later time points.

### Model validation on double-blind prospective cohort

**Fig. 4:**
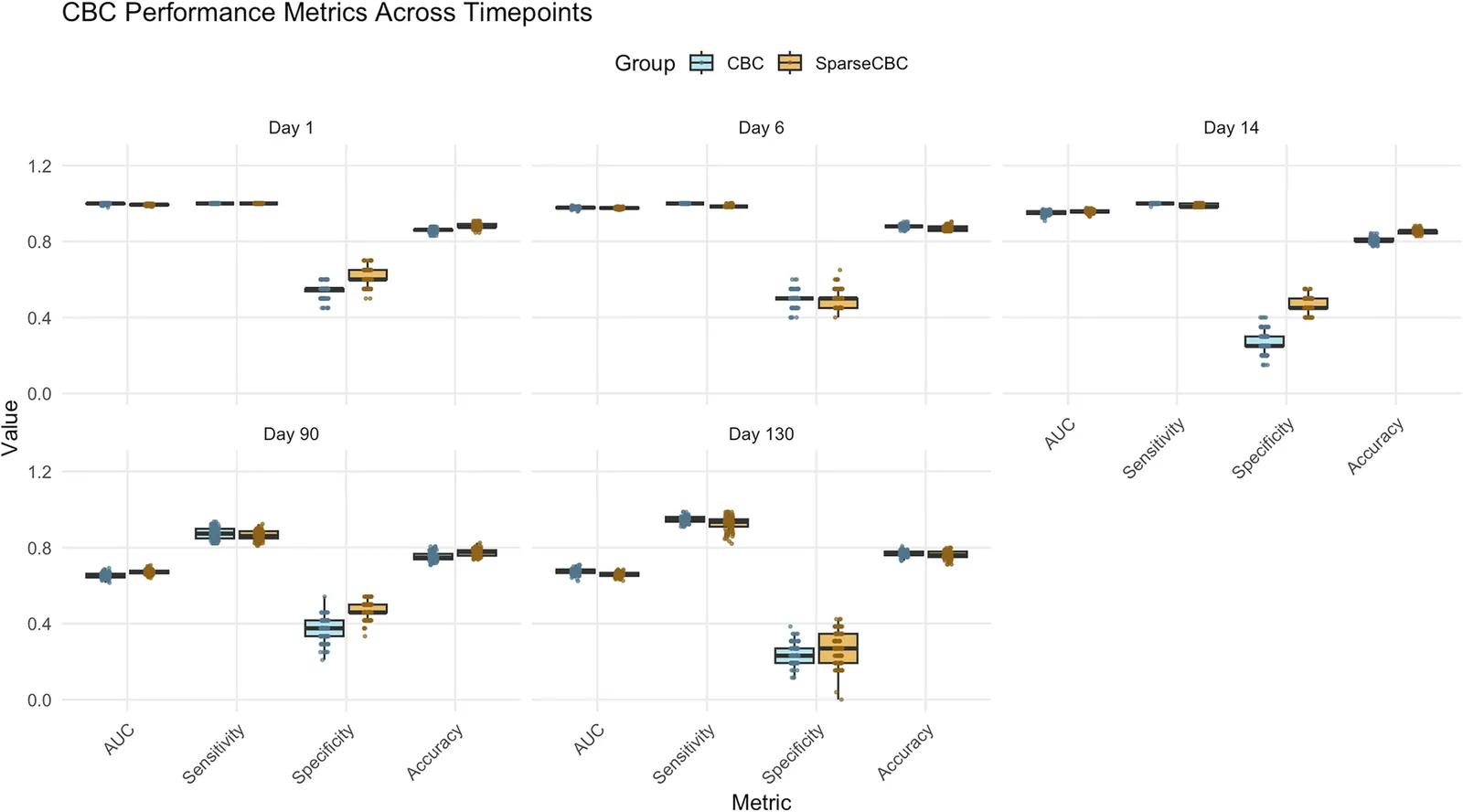
Prospective cohort model testing. Classification performance of the full CBC model and the Sparse CBC model was assessed at Days 1, 6, 14, 90 and 130 using AUC, sensitivity, specificity and accuracy. Boxplots show the distribution of the model performance values, with all individual data points overlaid *n* = 431. The *p* values obtained using two-sided Mann-Whitney U tests are provided in Table S5. Box and whisker plots indicate median, 25 and 75th percentiles, and individual samples beyond these limits.

In this step, the performance of random forest models on both original CBC and Sparse CBC was further prospectively and double-blindly validated. Specifically, we used all random forest models (100 models for original CBC and another 100 models for Sparse CBC) constructed during bootstrapping evaluation to form an assembled classifier for original CBC and Sparse CBC, respectively. The evaluation on double-blind prospective cohort clearly indicates that Sparse CBC model (AUC: 0.846 [95% CI: 0.845, 0.847]; Accuracy: 0.819 [95% CI: 0.817, 0.821]; Sensitivity: 0.944 [0.941, 0.947]; Specificity: 0.455 [0.455, 0.464]) consistently outperforms (*p* < 0.05) original CBC model (AUC: 0.845 [95% CI: 0.844, 0.846]; Accuracy: 0.807 [95% CI: 0.805, 0.810]; Sensitivity: 0.956 [95% CI: 0.953, 0.960]; Specificity: 0.373 [95% CI: 0.373, 0.382]) for the prediction of irradiation status (Fig. S3). The Sparse CBC model consistently outperforms the original CBC model at later time points after exposure, with this advantage becoming apparent from day 14 post-exposure onwards, as shown in Fig. 4 (Table S5). In contrast, at earlier time points, the Sparse CBC model performs less accurately than the original CBC model, particularly at 1-day post-exposure. This pattern is observed under internal resampling within the retrospective training cohort and is independently confirmed in the double-blind prospective validation cohort (Figs. 3, 4). At 6-days post-exposure, the two models exhibit similar performance, as illustrated in Figs. 3, 4. Overall, the Sparse CBC model demonstrates a stronger performance across a broader range of time points, especially at later stages after exposure, compared to the original CBC model. This suggests that the Sparse CBC model is more effective at capturing radiation-induced changes over time, making it a more reliable tool for biodosimetry at later time points.

### Model validation on population-based CC cohort

**Fig. 5:**
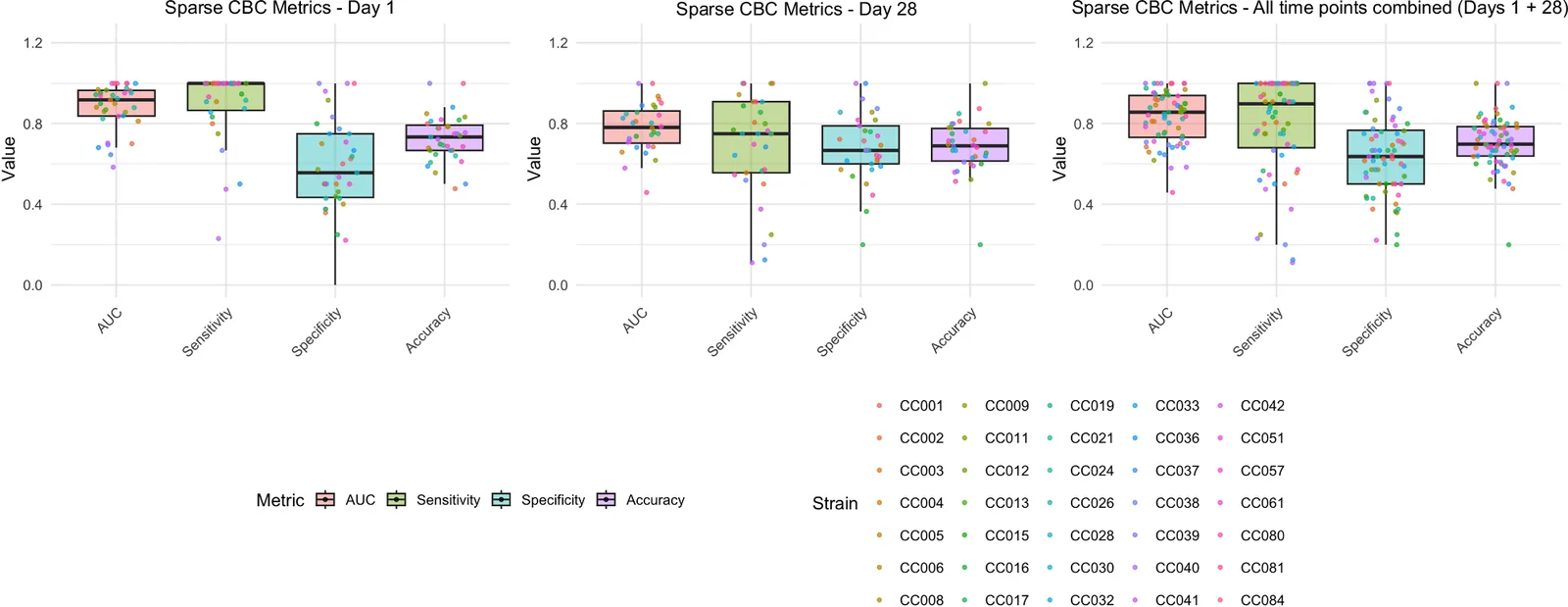
Genetically diverse cohort model testing. Classification performance of the Sparse CBC model on the CC cohort was evaluated at Day 1 (*n* = 799), Day 28 (*n* = 921) and using both timepoints combined (*n* = 1720), as measured by AUC, sensitivity, specificity and accuracy. Boxplots show the distribution of performance values across CC strains, with individual strain-level data points overlaid and color-coded by strain. Box and whisker plots indicate median, 25 and 75th percentiles, and individual samples beyond these limits.

Building on the validated performance of the Sparse CBC technique, we further assessed the robustness and generalizability of the pre-trained Sparse CBC model using a population-based CC cohort^14^. Applying the existing random forest models, we demonstrated that the Sparse CBC approach enables accurate prediction of radiation exposure across genetically diverse mouse strains. As observed in the retrospective and prospective cohorts, prediction accuracy was higher at 1-day post-exposure (Fig. 5A) compared to 28 days post-exposure (Fig. 5B). When combining both time points, overall model performance in the CC cohort remained strong, with an AUC of 0.786 [95% CI: 0.738, 0.833], accuracy of 0.691 [95% CI: 0.688, 0.713], sensitivity of 0.733 [95% CI: 0.642, 0.818], and specificity of 0.644 [95% CI: 0.588, 0.700] (Fig. 5C).

**Fig. 6:**
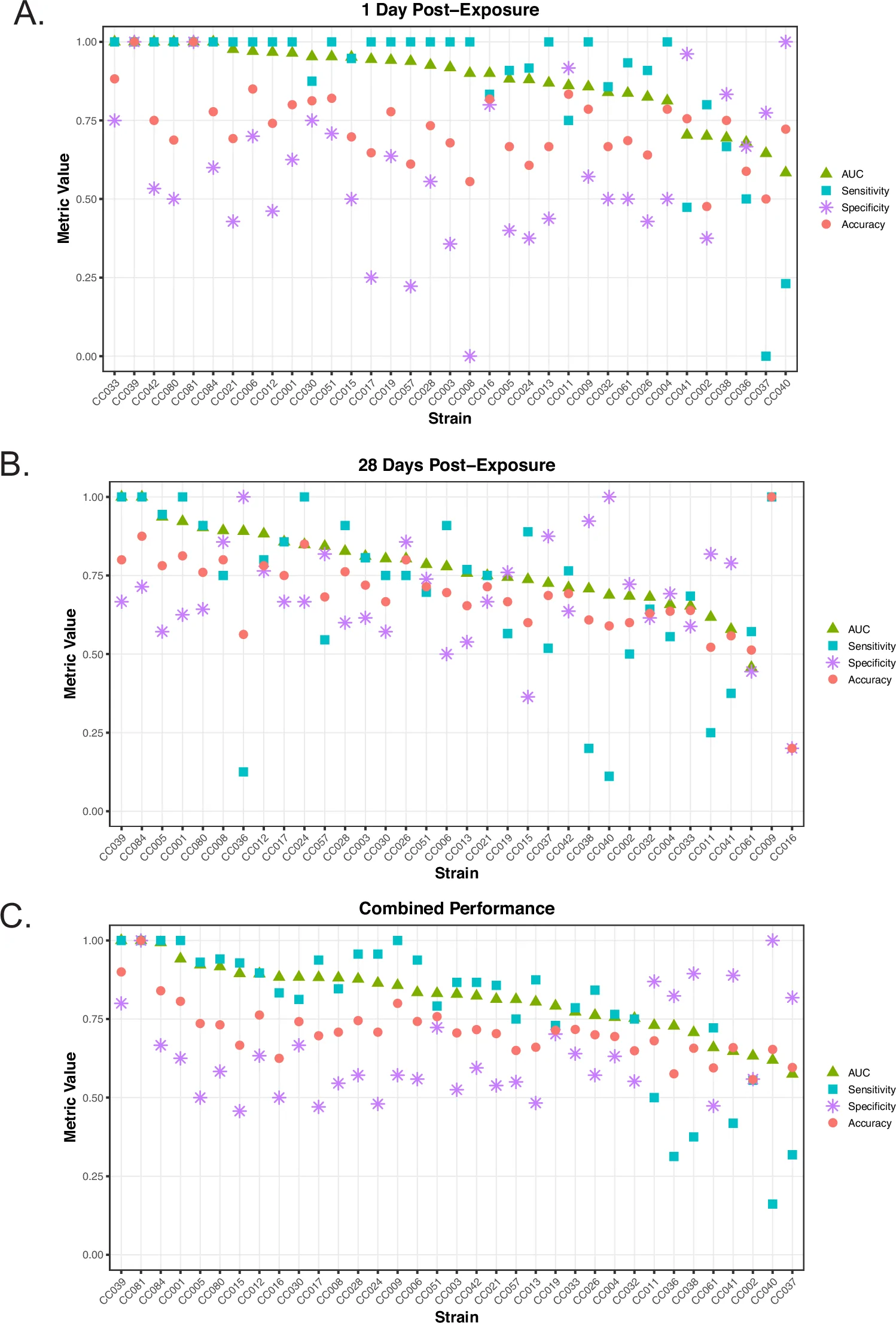
Individual strain performance in the genetically diverse cohort. **A** One day post-exposure. **B** Twenty-eight days post-exposure. **C** Combined performance metrics across timepoints. Plots show strain-level AUC, sensitivity, specificity and accuracy for individual CC strains in the genetically diverse cohort.

As expected, predictive performance varied substantially across different time points and CC strains (Fig. 6A–C), highlighting the critical influence of both genetic background and time post-exposure. These results emphasize that the accuracy and reliability of radiation exposure prediction models are shaped by the genetic context and temporal dynamics of the hematologic response. Therefore, to develop a broadly applicable and high-performing predictive model, it is essential to incorporate diverse genetic profiles and appropriately timed data. This approach will enhance model robustness and extend its utility across heterogeneous biological and exposure scenarios.

### Radiation exposure score

**Fig. 7:**
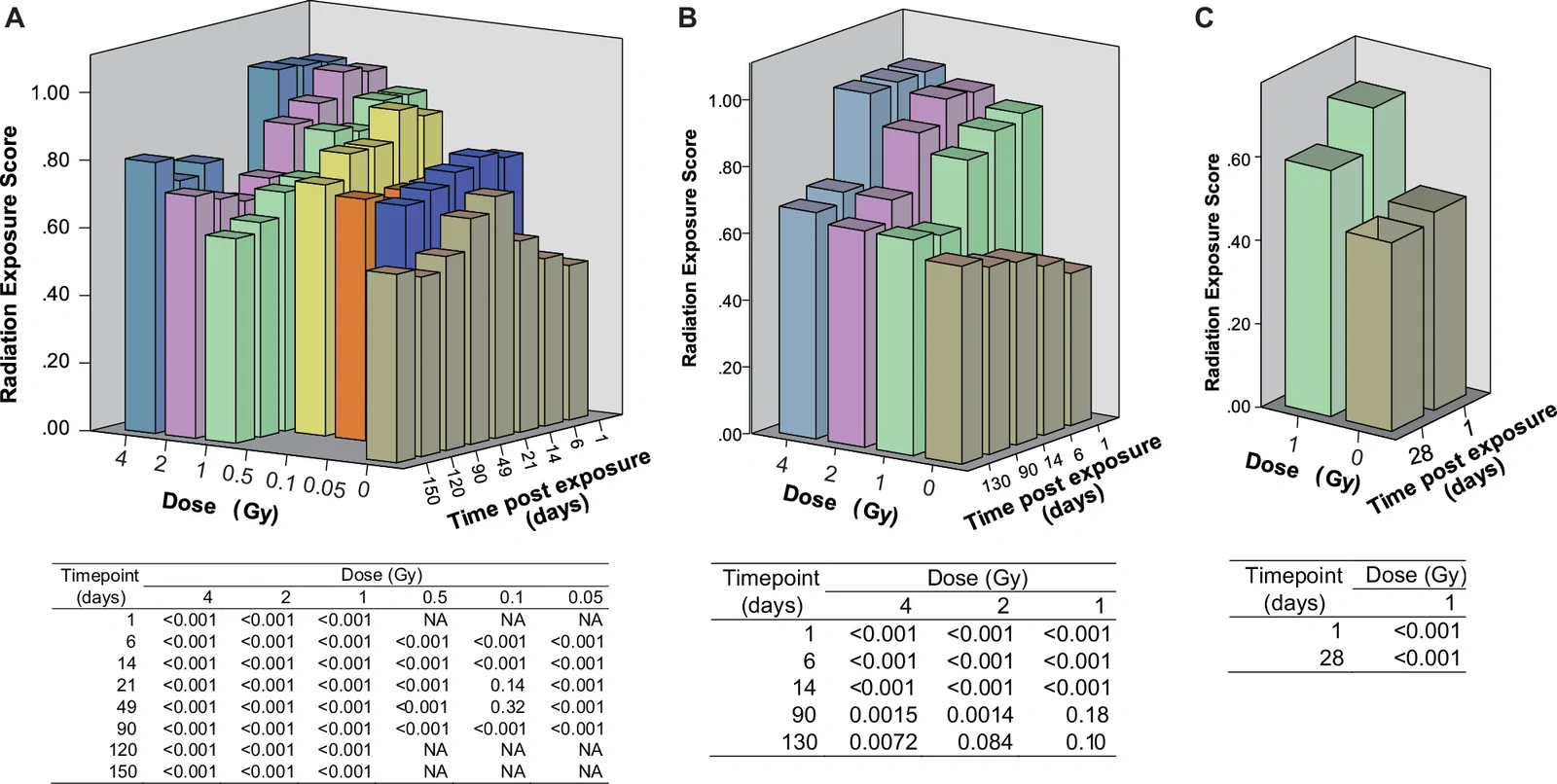
Radiation exposure score. **A** Radiation Exposure Score for retrospective cohort. **B** Radiation Exposure Score for prospective cohort. **C** Radiation Exposure Score in genetically diverse CC cohort. All p-values were obtained from generalized linear models by testing the treatment effect using two-sided Wald tests. No adjustment was applied for multiple comparisons.

We developed a Radiation Exposure Score (see methods) at dose-time point combinations post-exposure (Fig. 7). Radiation Exposure Scores exhibit a clear dose-dependent response, with higher doses correlating with higher scores. Notably, these scores are also significantly modulated by the time elapsed since radiation treatment, demonstrating dynamic temporal changes across all analyzed cohorts.

## Discussion

Searching for reliable biosignatures of ionizing radiation exposure remains a critical challenge in radiation research. In this study, we developed and validated an AI-based predictive model using CBC data to detect radiation exposure. By applying a Sparse CBC framework, we systematically evaluated the model across retrospective, prospective, and genetically diverse CC cohorts. Our results support the utility of CBC profiles not only as markers of hematopoietic damage following radiation but also as potential indicators of past exposure, including low-dose scenarios.

Although high AUC values indicate strong overall discriminative performance across decision thresholds, specificity values reported here were calculated using a fixed predicted probability cutpoint of 0.5, selected a priori as an unbiased decision boundary without post hoc optimization. Accordingly, sensitivity and specificity reflect performance at a single operating point rather than an optimized trade-off.

Assessing the effects of low-dose radiation remains particularly challenging, but our findings demonstrate that such exposures leave detectable hematologic signatures that can be captured using the Sparse CBC model. While hematological changes following radiation exposure have been extensively studied and form the basis of validated algorithms for assessing radiation dose and injury severity in life-threatening exposures, fewer studies have systematically characterized hematologic responses in the low-dose, sublethal exposure range, where signals may be more subtle and context-dependent^11,24^. This variability may reflect differences in genetic background and timing of measurement. Radiation damages the bone marrow, leading to reductions in red blood cells, white blood cells, and platelets, which manifest clinically if the dose is high. However, recovery kinetics vary by cell type and strain. We observed that genetic background significantly modulates hematologic responses to radiation, consistent with prior evidence highlighting its role in radiation susceptibility^14^. Our data also show that blood cell recovery after exposure typically begins within 2–4 weeks. As shown in this and our previous studies, the time for blood cells to recover depends on the type of blood cell and the genetic background, potentially accounting for discrepancies observed in prior studies.

The discovery of effective biomarkers is critical for assessing health and disease. Consequently, biomarkers play an important role in advancing the development of radiation biodosimetry tools that aid in assessing radiation exposure levels and determining the extent of radiation-induced injury. Model systems have been widely used to develop biomarkers for radiation biodosimetry. However, many studies have identified ionizing radiation biomarkers using only a single mouse strain. This study further demonstrates that the predictive model established from two strains of mice (C57BL/6 J and BALB/cJ) has lower efficiency in detecting radiation exposure in a population-based CC mouse model. Therefore, developing biomarkers for detecting radiation exposure should consider covariates, such as genetic backgrounds.

AI/ML have been extensively applied in biomarker research across various modalities^25–28^. Notably, unsupervised sparse learning has demonstrated significant power and utility in identifying patterns and signatures for both environmental exposure studies^27^ and precision oncology^25^. The application of unsupervised sparse learning on CBC phenotypes facilitates the identification of combinatorial CBC signatures that capture the intrinsic patterns in CBC phenotypes before and after low-dose radiation exposure and thus contribute to accurate prediction of historic low-dose radiation exposure that significantly exceeds the performance of classic machine learning models (i.e., random forest), specifically at 14 days post-exposure and beyond.

In conclusion, our comprehensive analysis reveals that an individual’s response to radiation is intricately linked to their unique genetic background and the time elapsed since exposure. This complex interplay underscores the importance of considering both genetic variability and temporal dynamics when developing biomarkers for biodosimetry. As the field evolves, future research must incorporate these variables to develop accurate, reliable, and generalizable tools for exposure assessment. By acknowledging these dimensions, we can move toward more personalized approaches that improve triage, medical management, and long-term monitoring of exposed individuals. Furthermore, by incorporating sparse feature selection and accounting for genetic variability, our approach lays the foundation for scalable, interpretable biodosimetry tools suitable for real-world, population-level applications.

## Supplementary information

Supplemental Material

Description of Additional Supplementary Data

Dataset 1

Dataset 2

Dataset 3

Dataset 4

## Data Availability

Data supporting the findings of this study are provided in the Supplementary Data 1-3 of this manuscript. Figure 2 is generated from Supplementary Data 1 and 2. Figure 3 is generated Supplementary Data 1; Fig. 4 is generated from Supplementary Data 2; Fig. 5 is generated from Supplementary Data 3; Fig. 6 is generated from Supplementary Data 3; Fig. 7 is generated from Supplementary Data 1–3 and the scores generated are included in Supplementary Data 4.

## Code availability

Source code and the corresponding machine learning models can be accessed on Zenodo at https://zenodo.org/records/20042951^29^.
