# Supplementary material for: Identifying predictive hematological biomarkers for radiation exposure by machine learning in mouse models
Source: Commun Med (Lond). 2026 Jun 19;6:516. doi: 10.1038/s43856-026-01690-8 (PMC13639052; doi:10.1038/s43856-026-01690-8)
Supplement: Supplementary file 1 [file 43856_2026_1690_MOESM1_ESM.pdf]

Table S1. Retrospective cohort characteristics

	BALB/cJ (N=578)	C57BL/6J (N=573)	Overall (N=1151)
**Sex**			
Female	396 (68.5%)	403 (70.3%)	799 (69.4%)
Male	182 (31.5%)	170 (29.7%)	352 (30.6%)
**Dosage (Gy)**			
0	158 (27.3%)	143 (25.0%)	301 (26.2%)
0.05	0 (0%)	45 (7.9%)	45 (3.9%)
0.1	53 (9.2%)	45 (7.9%)	98 (8.5%)
0.5	0 (0%)	43 (7.5%)	43 (3.7%)
1	135 (23.4%)	129 (22.5%)	264 (22.9%)
2	135 (23.4%)	81 (14.1%)	216 (18.8%)
4	97 (16.8%)	87 (15.2%)	184 (16.0%)
**Days post exposure**			
1	72 (12.5%)	61 (10.6%)	133 (11.6%)
6	106 (18.3%)	90 (15.7%)	196 (17.0%)
14	104 (18.0%)	86 (15.0%)	190 (16.5%)
21	52 (9.0%)	40 (7.0%)	92 (8.0%)
49	29 (5.0%)	52 (9.1%)	81 (7.0%)
90	76 (13.1%)	102 (17.8%)	178 (15.5%)
120	71 (12.3%)	71 (12.4%)	142 (12.3%)
150	68 (11.8%)	71 (12.4%)	139 (12.1%)

Table S2. Prospective cohort characteristics

	BALB/cJ (N=224)	C57BL/6J (N=207)	Overall (N=431)
**Sex**			
Female	113 (50.4%)	103 (49.8%)	216 (50.1%)
Male	111 (49.6%)	104 (50.2%)	215 (49.9%)
**Dosage (Gy)**			
0	57 (25.4%)	53 (25.6%)	110 (25.5%)
1	59 (26.3%)	55 (26.6%)	114 (26.5%)
2	50 (22.3%)	47 (22.7%)	97 (22.5%)
4	58 (25.9%)	52 (25.1%)	110 (25.5%)
**Days post exposure**			
1	40 (17.9%)	25 (12.1%)	65 (15.1%)
6	41 (18.3%)	42 (20.3%)	83 (19.3%)
14	38 (17.0%)	38 (18.4%)	76 (17.6%)
90	52 (23.2%)	51 (24.6%)	103 (23.9%)
130	53 (23.7%)	51 (24.6%)	104 (24.1%)

Table S3. Collaborative Cross cohort characteristics

Strain	Males	Females	0Gy	1Gy	Day1	Day28
CC001 (n=31)	16 (51.6%)	15 (48.4%)	16 (51.6%)	15 (48.4%)	15 (48.4%)	16 (51.6%)
CC002 (n=61)	24 (39.3%)	37 (60.7%)	34 (55.7%)	27 (44.3%)	21 (34.4%)	40 (65.6%)
CC003 (n=85)	45 (52.9%)	40 (47.1%)	40 (47.1%)	45 (52.9%)	28 (32.9%)	57 (67.1%)
CC004 (n=36)	17 (47.2%)	19 (52.8%)	19 (52.8%)	17 (47.2%)	14 (38.9%)	22 (61.1%)
CC005 (n=53)	24 (45.3%)	29 (54.7%)	24 (45.3%)	29 (54.7%)	21 (39.6%)	32 (60.4%)
CC006 (n=66)	42 (63.6%)	24 (36.4%)	34 (51.5%)	32 (48.5%)	20 (30.3%)	46 (69.7%)
CC008 (n=24)	12 (50%)	12 (50%)	11 (45.8%)	13 (54.2%)	9 (37.5%)	15 (62.5%)
CC009 (n=15)	10 (66.7%)	5 (33.3%)	7 (46.7%)	8 (53.3%)	14 (93.3%)	1 (6.7%)
CC011 (n=47)	18 (38.3%)	29 (61.7%)	23 (48.9%)	24 (51.1%)	24 (51.1%)	23 (48.9%)
CC012 (n=59)	28 (47.5%)	31 (52.5%)	30 (50.8%)	29 (49.2%)	27 (45.8%)	32 (54.2%)
CC013 (n=53)	26 (49.1%)	27 (50.9%)	29 (54.7%)	24 (45.3%)	27 (50.9%)	26 (49.1%)
CC015 (n=63)	33 (52.4%)	30 (47.6%)	35 (55.6%)	28 (44.4%)	43 (68.3%)	20 (31.7%)
CC016 (n=16)	8 (50%)	8 (50%)	10 (62.5%)	6 (37.5%)	11 (68.8%)	5 (31.2%)
CC017 (n=33)	18 (54.5%)	15 (45.5%)	17 (51.5%)	16 (48.5%)	17 (51.5%)	16 (48.5%)
CC019 (n=84)	45 (53.6%)	39 (46.4%)	47 (56%)	37 (44%)	36 (42.9%)	48 (57.1%)
CC021 (n=27)	14 (51.9%)	13 (48.1%)	13 (48.1%)	14 (51.9%)	13 (48.1%)	14 (51.9%)
CC024 (n=48)	27 (56.2%)	21 (43.8%)	25 (52.1%)	23 (47.9%)	28 (58.3%)	20 (41.7%)
CC026 (n=40)	20 (50%)	20 (50%)	21 (52.5%)	19 (47.5%)	25 (62.5%)	15 (37.5%)

Strain	Males	Females	0Gy	1Gy	Day1	Day28
CC028 (n=51)	30 (58.8%)	21 (41.2%)	28 (54.9%)	23 (45.1%)	30 (58.8%)	21 (41.2%)
CC030 (n=31)	17 (54.8%)	14 (45.2%)	15 (48.4%)	16 (51.6%)	16 (51.6%)	15 (48.4%)
CC032 (n=57)	46 (80.7%)	11 (19.3%)	29 (50.9%)	28 (49.1%)	30 (52.6%)	27 (47.4%)
CC033 (n=53)	33 (62.3%)	20 (37.7%)	25 (47.2%)	28 (52.8%)	17 (32.1%)	36 (67.9%)
CC036 (n=33)	17 (51.5%)	16 (48.5%)	17 (51.5%)	16 (48.5%)	17 (51.5%)	16 (48.5%)
CC037 (n=99)	43 (43.4%)	56 (56.6%)	55 (55.6%)	44 (44.4%)	48 (48.5%)	51 (51.5%)
CC038 (n=35)	23 (65.7%)	12 (34.3%)	19 (54.3%)	16 (45.7%)	12 (34.3%)	23 (65.7%)
CC039 (n=10)	10 (100%)	0 (0%)	5 (50%)	5 (50%)	5 (50%)	5 (50%)
CC040 (n=75)	38 (50.7%)	37 (49.3%)	44 (58.7%)	31 (41.3%)	36 (48%)	39 (52%)
CC041 (n=88)	51 (58%)	37 (42%)	45 (51.1%)	43 (48.9%)	45 (51.1%)	43 (48.9%)
CC042 (n=67)	39 (58.2%)	28 (41.8%)	37 (55.2%)	30 (44.8%)	28 (41.8%)	39 (58.2%)
CC051 (n=95)	43 (45.3%)	52 (54.7%)	47 (49.5%)	48 (50.5%)	39 (41.1%)	56 (58.9%)
CC057 (n=40)	22 (55%)	18 (45%)	20 (50%)	20 (50%)	18 (45%)	22 (55%)
CC061 (n=74)	38 (51.4%)	36 (48.6%)	38 (51.4%)	36 (48.6%)	35 (47.3%)	39 (52.7%)
CC080 (n=41)	20 (48.8%)	21 (51.2%)	24 (58.5%)	17 (41.5%)	16 (39%)	25 (61%)
CC081 (n=5)	5 (100%)	0 (0%)	2 (40%)	3 (60%)	5 (100%)	0 (0%)
CC084 (n=25)	15 (60%)	10 (40%)	12 (48%)	13 (52%)	9 (36%)	16 (64%)
**Total**	**Male**	**Female**	**0Gy**	**1Gy**	**Day1**	**Day28**
1,720	917 (53.3%)	803 (46.7%)	897 (52.2%)	823 (47.8%)	799 (46.5%)	921 (53.5%)

Table S4. Performance comparison between CBC and SparseCBC in the retrospective cohort, where p values were obtained using Wilcoxon non-parametric tests.

Timepoint (day)	AUC	Accuracy	Sensitivity	Specificity
1	<0.001	0.074	1.000	0.331
6	0.022	0.649	0.952	0.704
14	<0.001	<0.001	0.227	<0.001
21	0.001	0.248	0.221	0.425
49	0.003	0.003	0.040	0.229
90	<0.001	0.179	0.719	<0.001
120	<0.001	<0.001	<0.001	<0.001
150	<0.001	<0.001	<0.001	0.028

Table S5. Performance comparison between CBC and SparseCBC in the prospective cohort, where p values were obtained using Wilcoxon non-parametric tests.

Timepoint (Day)	AUC	Accuracy	Sensitivity	Specificity
1	<0.001	<0.001	N/A	<0.001
6	0.00295	<0.001	<0.001	0.555
14	<0.001	<0.001	<0.001	<0.001
90	<0.001	<0.001	0.0557	<0.001
130	<0.001	0.00394	<0.001	0.00314

Figure S1. Heatmaps and Relative Lymphocyte Counts and Neutrophil Percents as a function of dose

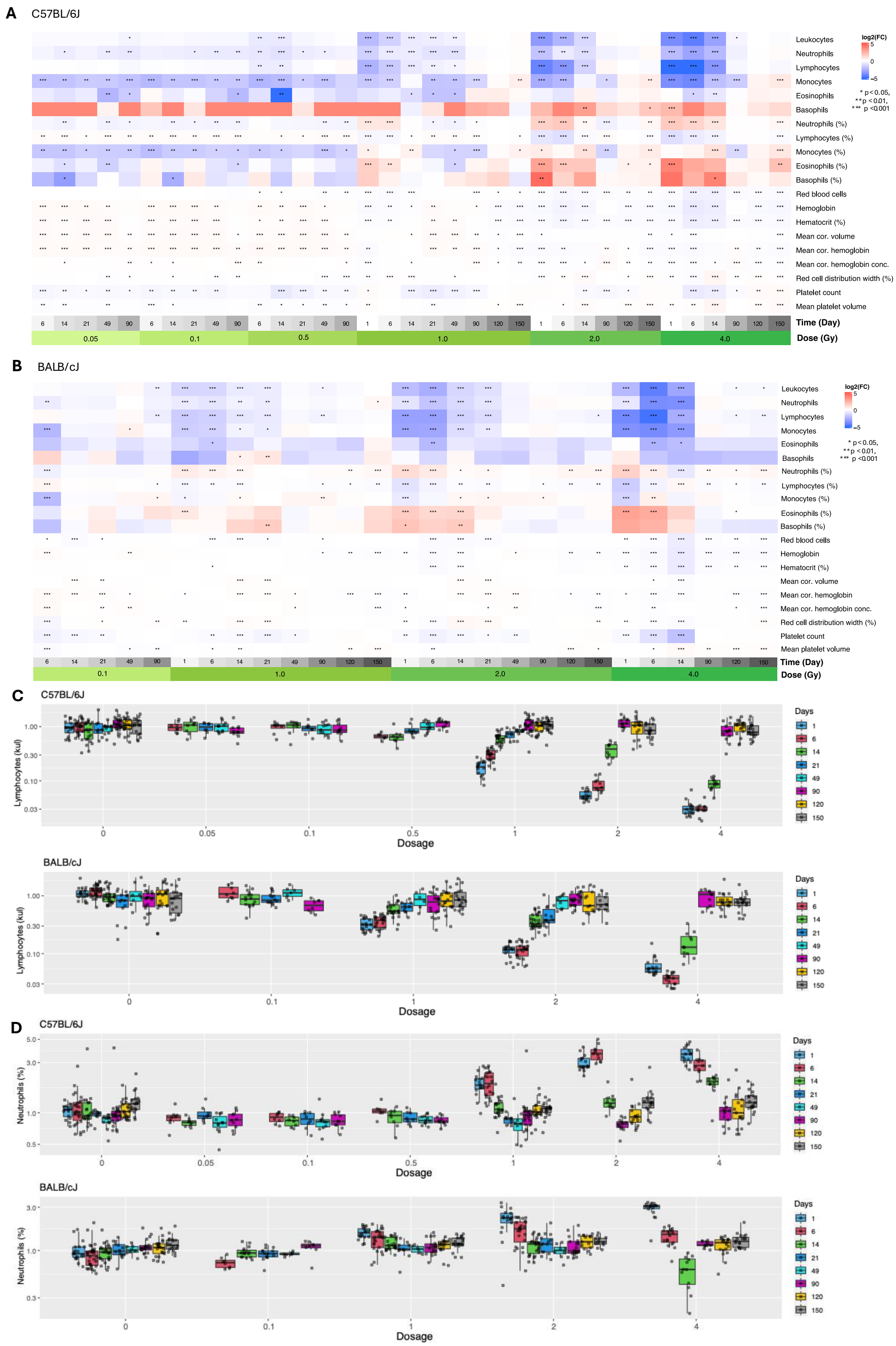

Figure S1. Heatmaps and Relative Lymphocyte Counts and Neutrophil Percents as a function of dose A) Heatmap of changes in CBC parameters in C57BL/6J CBC counts post-exposure in the Retrospective Cohort. Dose is indicated on the bottom in green (0.05-4Gy) and time post exposure in gray (1-150 days). Significance for each metric is indicated in the heat graph with * p<0.05, ** p<0.01, *** p<0.001. B) Heat map of changes in CBC parameters in Balb/cJ CBC counts post-exposure in the Retrospective Cohort. Dose is indicated on the bottom in green (0.1-4Gy) and time post exposure in gray (1-150 days). Significance for each metric is indicated in the heat graph with * p<0.05, ** p<0.01, *** p<0.001. C) Relative lymphocyte counts (kul) in C57BL/6J (top) and Balb/cJ (bottom) grouped by exposure dose. D) Relative neutrophil % in C57BL/6J (top) and Balb/cJ (bottom) grouped by exposure dose.

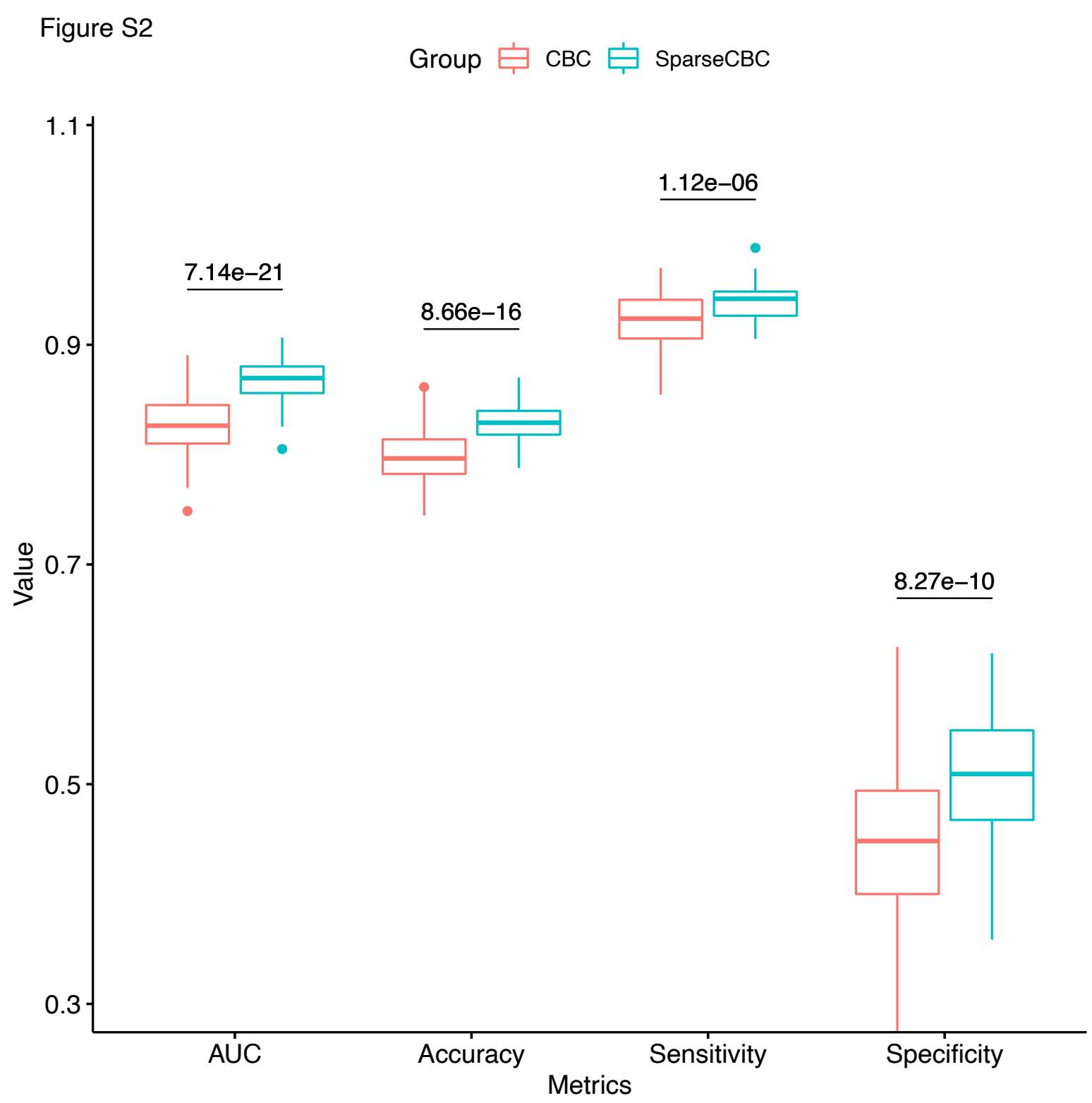

Figure S2. Model Construction Retrospective Cohort. Boxplots show AUC, sensitivity, specificity and accuracy for the full CBC and Sparse CBC models for all time points post exposure combined. The p values obtained using Wilcoxon non-parametric tests.

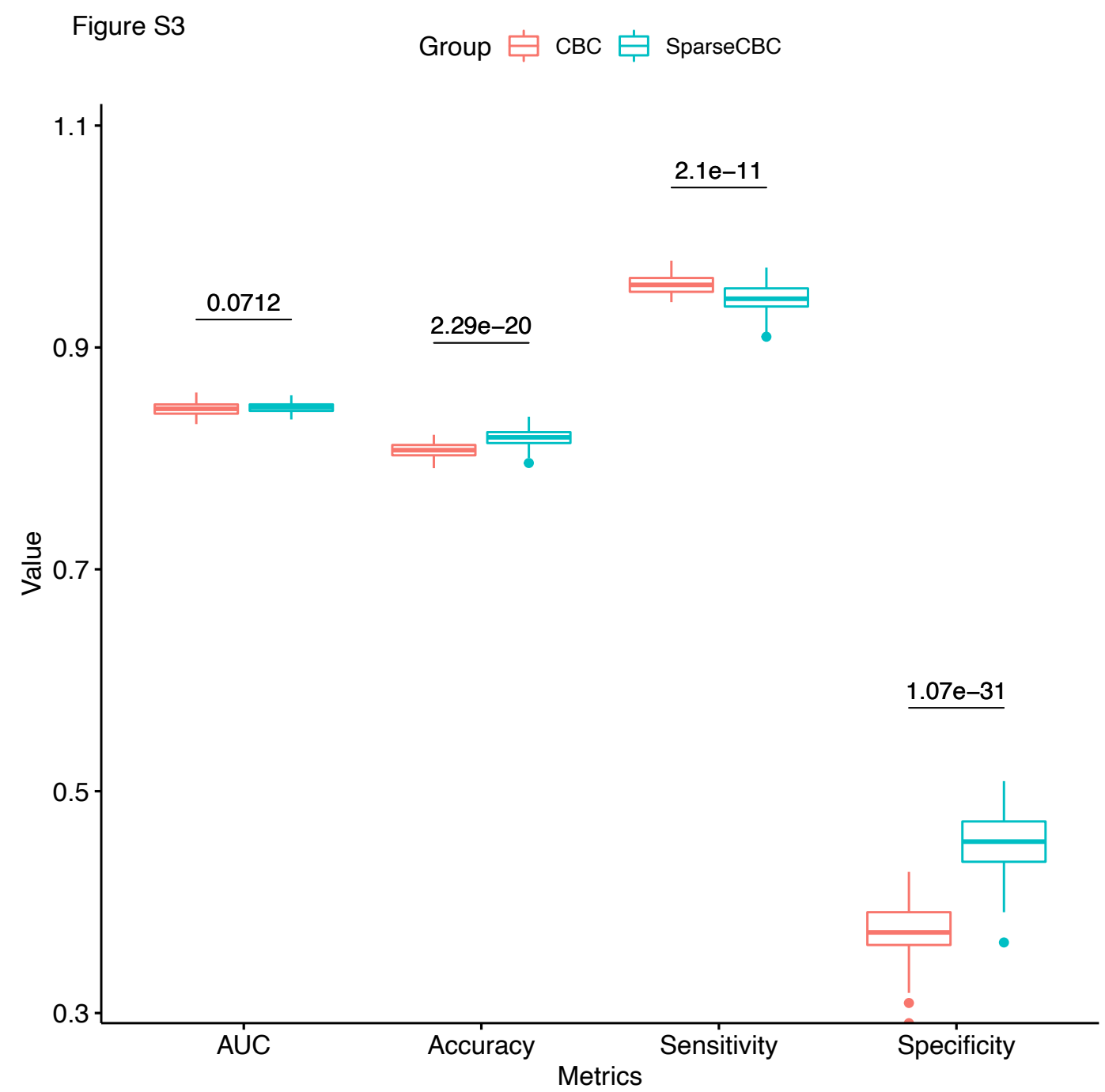

Figure S3. Prospective Cohort Model Testing. Classification performance of the full CBC model and the Sparse CBC model was assessed for all time points post exposure combined using AUC, sensitivity, specificity and accuracy. Boxplots show the distribution of the model performance values. The p values obtained using Wilcoxon non-parametric tests.
