# Supplementary material for: Identifying predictive hematological biomarkers for radiation exposure by machine learning in mouse models
Source: Commun Med (Lond). 2026 Jun 19;6:516. doi: 10.1038/s43856-026-01690-8 (PMC13639052; doi:10.1038/s43856-026-01690-8)
Supplement: Supplementary file 2 [file 43856_2026_1690_MOESM2_ESM.docx]

Description of additional supplementary file

File name: Dataset 1

Description: Data 1: Retrospective Training Cohort CBC Records

File name: Dataset 2

Description: Data 2: Prospective Cohort CBC Records

File name: Dataset 3

Description: Data 3. CBC records of 1720 CC mice representing 35 individual strains at 24 hours and four weeks after sham or X-ray exposure.

File name: Dataset 4

Description: Radiation exposure score in prospective cohort
